# Pre-Altitude Serum Ferritin Levels and Daily Oral Iron Supplement Dose Mediate Iron Parameter and Hemoglobin Mass Responses to Altitude Exposure

**DOI:** 10.1371/journal.pone.0135120

**Published:** 2015-08-11

**Authors:** Andrew D. Govus, Laura A. Garvican-Lewis, Chris R. Abbiss, Peter Peeling, Christopher J. Gore

**Affiliations:** 1 Centre for Exercise and Sports Science Research, School of Exercise and Health Science, Edith Cowan University, Joondalup, WA, Australia; 2 School of Sport Science, Exercise & Health, University of Western Australia, Crawley, WA, Australia; 3 Department of Physiology, Australian Institute of Sport, Bruce, ACT, Australia; 4 Exercise Physiology Laboratory, Flinders University, Bedford Park, SA, Australia; 5 Research Institute for Sport and Exercise, University of Canberra, Bruce, ACT, Australia; The Pennsylvania State University Hershey Medical Center, UNITED STATES

## Abstract

**Purpose:**

To investigate the influence of daily oral iron supplementation on changes in hemoglobin mass (Hb_mass_) and iron parameters after 2–4 weeks of moderate altitude exposure.

**Methods:**

Hematological data collected from 178 athletes (98 males, 80 females) exposed to moderate altitude (1,350–3,000 m) were analysed using linear regression to determine how altitude exposure combined with oral iron supplementation influenced Hb_mass_, total iron incorporation (TII) and blood iron parameters [ferritin and transferrin saturation (TSAT)].

**Results:**

Altitude exposure (mean ± *s*: 21 ± 3 days) increased Hb_mass_ by 1.1% [-0.4, 2.6], 3.3% [1.7, 4.8], and 4.0% [2.0, 6.1] from pre-altitude levels in athletes who ingested nil, 105 mg and 210 mg respectively, of oral iron supplement daily. Serum ferritin levels decreased by -33.2% [-46.9, -15.9] and 13.8% [-32.2, 9.7] from pre-altitude levels in athletes who supplemented with nil and 105 mg of oral iron supplement daily, but increased by 36.8% [1.3, 84.8] in athletes supplemented with 210 mg of oral iron daily. Finally, athletes who ingested either 105 mg or 210 mg of oral iron supplement daily had a greater TII compared with non-supplemented athletes (0 *versus* 105 mg: effect size (*d*) = -1.88 [-2.56, -1.17]; 0 *versus* 210 mg: effect size (*d*) = -2.87 [-3.88, -1.66]).

**Conclusion:**

Oral iron supplementation during 2–4 weeks of moderate altitude exposure may enhance Hb_mass_ production and assist the maintenance of iron balance in some athletes with low pre-altitude iron stores.

## Introduction

Prolonged moderate altitude exposure (i.e. several weeks at >2,000 m) enhances oxygen transport and utilization by stimulating hematological [i.e. increased hemoglobin mass (Hb_mass_)] and non-hematological (i.e. increased skeletal muscle buffer capacity, mitochondrial density, glycolytic and oxidative enzyme concentration) adaptations [1]. Substantial inter- and intra-individual variability exists in the magnitude of the Hb_mass_ response to prolonged moderate altitude exposure [2], owing to several factors including the hypoxic dose [3], variations in kidney erythropoietin production during early adaptation [4], as well as injury and illness [5].

Sufficient iron stores are required to support an hypoxic-mediated increase in heme synthesis and iron-dependent enzyme production during prolonged altitude exposure. Indeed, altitude exposure is associated with a three- to five-fold increase in erythropoiesis, with erythroid iron uptake approaching 100% of its maximal capacity during the first few days of adaptation [6]. However, low pre-altitude iron stores, or an inability to rapidly mobilise iron from reticuloendothelial macrophages may reduce the amount of iron available to support hematological and non-hematological adaptations during initial altitude exposure. For example, red cell volume did not improve following four weeks of moderate altitude (2,500 m) exposure in nine non-iron supplemented, iron deficient runners (pre-altitude serum ferritin: 15 ± 3 μg·L^-1^), suggesting athletes require sufficient pre-altitude serum ferritin levels to support accelerated erythropoiesis at altitude [7]. In contrast, Ryan et al. [8] reported that 7 of 9 non-iron supplemented female subjects exposed to 5,000 m for 16 days increased their Hb_mass_ despite low pre-altitude serum ferritin levels (28.9 ± 15.5 μg·L^-1^). Moreover, these authors reported a weak correlation (*r* = 0.33, *p* = 0.16) between pre-altitude serum ferritin levels and the percentage change in Hb_mass_ after 16 days of high altitude exposure. Therefore, the influence of pre-altitude ferritin levels on the Hb_mass_ response to prolonged altitude exposure is currently unclear.

To date, there are no clear iron supplementation guidelines for athletes planning to undertake moderate altitude exposure. Currently, it is thought that raising an athlete’s pre-altitude iron stores via oral iron supplementation in the weeks before altitude exposure may assist them to maintain a healthy iron balance when training at altitude [9]. Furthermore, although oral iron supplementation guidelines at sea level recommend providing oral iron supplements to athletes with serum ferritin levels <35 μg·L^-1^ [10], athletes with otherwise healthy serum ferritin levels (i.e. >35 μg·L^-1^) planning to undertake a period of altitude exposure, may also need to ingest oral iron supplements to provide sufficient iron for erythropoiesis and iron-dependent enzyme production. Furthermore, there is some evidence that iron supplementation may alleviate the symptoms of altitude-related illnesses such as acute mountain sickness [11], and help to maintain cognitive function in hypoxia [12].

Relatively few studies have investigated the influence of oral iron supplementation on Hb_mass_ production in elite athletes undertaking moderate altitude exposure. In one study, oral iron supplementation (200 mg elemental iron daily) did not enhance the Hb_mass_ response relative to a placebo in 17 National level boxers exposed to moderate altitude (1,800 m) for 18 days [13]. Oral iron supplementation may have been unnecessary in this group since their pre-altitude serum ferritin levels were clinically normal (serum ferritin: 70 ± 23 μg·L^-1^) and the overall hypoxic dose was lower than that typically used during Live High: Train Low (LHTL) protocols (i.e. 21 days exposure to 3,000 m). However, male and female endurance athletes with serum ferritin levels <40 μg·L^-1^, or who are training for long periods at moderate (2,000–3000 m) or high altitudes (>3,000 m) may require oral iron supplementation to sustain accelerated erythropoiesis [9].

To this end, we modelled the Hb_mass_ and blood iron parameters [serum ferritin, and transferrin saturation (TSAT)] response to prolonged moderate altitude exposure in a large sample of well-trained athletes. Additionally, we investigated how different oral iron supplement doses moderated the Hb_mass_ and iron parameter responses in well-trained athletes.

## Methods

### Ethics Statement

The Human Ethics Committee at the Australian Institute of Sport granted ethical approval for each altitude exposure. Before each altitude exposure, the risks and benefits associated with participation were explained to each participant in full before written informed consent was obtained. Additionally, the Human Research Ethics Committee at Edith Cowan University granted ethical approval for data analysis.

### Participants

De-identified hematological data (iron parameters and Hb_mass_) from 178 athletes (males = 98, females = 80) who undertook altitude exposure at the Australian Institute of Sport (AIS) from 2006 to 2014 were extracted from the AIS medical records database. This sample represents the entire population of endurance athletes who undertook altitude exposure at the AIS during this time. Athletes were from one of seven sports, including; cycling (*n* = 60), rowing (*n* = 9), swimming (*n* = 2), triathlon (*n* = 5), distance running (*n* = 43), race walking (*n* = 39) and water polo (*n* = 20). Athletes were AIS scholarship holders and/or national team members who undertook altitude exposure as part of a training camp, or who attended the AIS for research purposes. Hence, some aspects of the combined data set presented here have been published previously [14–19].

Three females were diagnosed by a sports physician as having hereditary hemochromatosis and were removed at the data extraction phase and not included in the final data analysis. Athletes’ iron status was classified based on their pre-altitude exposure serum ferritin levels. Iron deficiency was categorised into three stages: Stage 1 (iron deficiency): serum ferritin <35 μg·L^-1^, TSAT >16%; Stage 2 (iron deficient erythropoiesis): serum ferritin <20 μg·L^-1^, TSAT >16%; Stage 3: serum ferritin <12 μg·L^-1^, TSAT <16% is normally associated with anemia [20].

Prior to altitude exposure, 21 athletes (5 males, serum ferritin: 30.1 ± 5.3 μg·L^-1^; 16 females, serum ferritin: 29.7 ± 3.4 μg·L^-1^) met the criteria for Stage 1 iron deficiency. Six athletes met the criteria for Stage 2 iron deficiency (2 males, serum ferritin: 17.4 ± 0.7 μg·L^-1^; 4 females, serum ferritin: 15.2 ± 3.7 μg·L^-1^) and one female athlete met the criteria for Stage 3 iron deficiency (serum ferritin: 11.8 μg·L^-1^).

### Altitude Training

Four altitude training camps employed a classic [Live High: Train High, (LHTH)] training approach and were conducted at either Stelvio Pass, Italy (~2,700 m, *n* = 5; [15]), St. Moritz, Switzerland (~1,850 m, *n* = 5), Perisher Valley, Australia (~1,720 m, *n* = 8) or Thredbo, Australia (~1,350 m, *n* = 13). The remaining altitude camps used a Live High: Train Low (LHTL) approach (*n* = 147) and were conducted in normobaric, altitude-training facility at the AIS [16], designed to simulate 3,000 m altitude. During LHTL camps, athletes were required to spend a minimum of 14 hours per day at altitude, whilst they trained near sea level (~600 m, Canberra, Australia). One LHTL group lived at 3,000 m but performed three training sessions per week at 2,200 m [19].

### Iron Supplementation

Iron supplements (Ferro Grad C, 325 mg ferrous sulphate & 1,000 mg ascorbic acid, delivering 105 mg elemental iron, Abbott Laboratories, Botany Bay, Australia) were administered to athletes one week prior to, and for the duration of, altitude exposure. The iron supplement dose administered during altitude exposure was based on athletes’ pre-altitude ferritin levels. Fifteen athletes (13 males, 2 females) were not iron supplemented because of high (i.e. >100 μg·L^-1^) pre-altitude serum ferritin levels [serum ferritin (mean ± *s*): 164 ± 35 μg·L^-1^]. Additionally, 144 athletes (82 males, 62 females) ingested one iron tablet per day (105 mg elemental iron; serum ferritin: 76 ± 32 μg·L^-1^). Finally, 19 athletes (3 males, 16 females) ingested two iron tablets per day (210 mg elemental iron; serum ferritin: 25 ± 7 μg·L^-1^).

### Hemoglobin Mass (Hb_mass_)

Hemoglobin mass was measured before and after altitude exposure via the optimised 2 min carbon monoxide (CO) rebreathing technique [21]. Briefly, this technique involved the rebreathing of a bolus of ~1.2 mL·kg^-1^ CO for 2 min. Capillary blood samples were taken before and 7 min after rebreathing CO, and measured five times using an OSM3 Hemoximeter (Radiometer, Copenhagen, Denmark) to determine the percentage change in carboxyhemoglobin (HbCO). Hemoglobin mass was then calculated from the change in %HbCO before and after CO rebreathing. The typical error of measurement for Hb_mass_ in our laboratory is ~1.8%, 90% CI [1.3, 2.2] [22].

### Iron Parameters

Blood samples were collected at rest from a forearm antecubital vein by a trained phlebotomist. Pre- and post-altitude iron parameters (serum ferritin and TSAT) were measured at the AIS using either a Hitachi 911 (Boehringer, GmbH, Mannaheim, Ingelheim, Germany) or a COBAS Integra 400 (Roche Diagnostics, Switzerland) via immunoturbidimetry [23], with the same analyser always used for each pre *versus* post comparison.

### Total Iron Incorporation

Total Iron Incorporation (TII) was calculated to determine total erythrocyte iron uptake (HII) and iron stored as ferritin during altitude exposure (SII) [24]. Here, TII = [(Δ Hb_mass_ (g) × 3.38 mg) + (Δ Ferritin (μg·L^-1^) × 8 mg)], where, 3.38 mg represents the typical iron content of Hb [25] and 8 mg represents the iron content of ferritin [26].

### Statistics

#### Linear Regression

The influence of altitude exposure on Hb_mass_ and iron parameters was modelled using a multiple regression in the R statistics programme [27]. Hb_mass_, serum ferritin, TSAT and were log-transformed before analysis, back transformed, and then expressed as a percentage difference from the specified reference level to improve interpretability. Data were first modelled to investigate the influence of the following covariates on the change in Hb_mass_ in response to prolonged moderate altitude exposure: sex (2 levels: male, female), altitude category (2 levels: natural, simulated), altitude duration (<2 weeks, 2–3 weeks, >3 weeks), altitude elevation (3 levels: 1,350–1,850 m, 2.600–2,800 m, 3,000 m) and sport (7 levels: race walking, distance running, road cycling, triathlon, rowing, swimming and waterpolo). There were no significant differences between the changes in Hb_mass_ or iron parameters following altitude exposure for sex and altitude category, thus these data were pooled during the final analysis. Furthermore, since the interaction between sport and oral iron supplement dose yielded a small sample size (*n* = 1 to 2), we chose to omit these covariates from the final model.

The percentage change in Hb_mass_ and serum iron parameters during altitude exposure was analysed via linear regression with daily oral iron supplement dose (3 levels: none, 105 mg, 210 mg) as the only model covariate. Results are reported as the percentage change from pre-altitude values, with 95% confidence intervals to denote the imprecision of the point-estimate.

#### Effect Sizes

The magnitudes of the change in pre-altitude *versus* post-altitude values for TII, HII and SII are expressed as a Cohen’s *d* effect size [28]. Effect sizes were interpreted using Cohen’s Scale for Effect Sizes [28] with the following qualitative descriptors: *trivial (0*.*0–0*.*2)*, *small (0*.*2–0*.*6)*, *moderate (0*.*6–1*.*2)*, *large (1*.*2–2*.*0)*, *very large (2*.*0–4*.*0)*. Additionally, the imprecision of the estimate was quantified using 95% confidence intervals. Since TII and SII included negative values, non-normality was addressed by adding 1,000 to these values before applying a Box-Cox (power) transformation [29].

## Results

Twenty-one athletes were classified as Stage 1 iron deficient (16 males, 5 females; serum ferritin: 29.8 ± 3.8 μg·L^-1^, TSAT: 26.7 ± 12.9%) and six athletes were classified as Stage 2 iron deficient (4 females, 2 males; serum ferritin: 15.9 ± 3.1 μg·L^-1^, TSAT: 27.2 ± 11.8%). We also classified athletes as hematological responders or non-responders based on a change in Hb_mass_ greater than the typical error of measurement for the CO rebreathing technique (i.e. 1.8%) [22]. Based on this criterion, 120 athletes (57 males, 63 females) athletes were classified as hematological responders and 58 athletes (35 males, 23 females) as non-responders.

### Hb_mass_ and Iron Parameter Response

The Hb_mass_ and iron parameter responses to moderate altitude exposure adjusted for oral iron supplement doses are presented in Table 1. Hb_mass_ increased from pre-altitude levels in athletes who ingested 105 mg and 210 mg of oral iron supplements (both *p* < 0.01). In comparison, Hb_mass_ did not increase significantly from pre-altitude levels following moderate altitude exposure in non-iron supplemented athletes (*p* = 0.14). ΔHb_mass_ was 2.1% [0.3, 4.0] and 2.9% [0.5, 5.3] higher in athletes who ingested 105 mg and 210 mg of oral iron per day compared with non-iron supplemented athletes. Serum ferritin decreased following moderate altitude exposure in non-iron supplemented athletes and those athletes who ingested 105 mg (both *p* < 0.01), but increased relative to pre-altitude levels in those athletes who ingested 210 mg of oral iron per day (*p* < 0.01). Finally, oral iron supplementation did not influence the ΔTSAT following moderate altitude exposure (*p* > 0.05).

**Table 1:** Parameter estimates (Est.) with 95% confidence intervals (CI) for the changes (Δ) in Hb_mass_, ferritin and transferrin saturation (TSAT) during prolonged moderate altitude exposure, when controlled for oral iron supplement dose.

Effect	Δ Hb_mass_(%)			Δ Ferritin (%)			Δ TSAT (%)		
Supplement Dose	Est.	95% CI	*n*	Est.	95% CI	*n*	Est.	95% CI	*n*
None	1.1	[-0.4, 2.6]	15	-33.2	[-46.9, -15.9]	10	-22.3	[-48.7, 17.8]	4
105 mg	3.3	[1.7, 4.8]	144	-13.8	[-32.2, 9.7]	97	-6.8	[-6.8, 42.6]	75
210 mg	4.0	[2.0, 6.1]	19	36.8	[1.3, 84.8]	15	10.9	[-31.6, 80.0]	11

### Total Iron Incorporation

**Fig 1:**
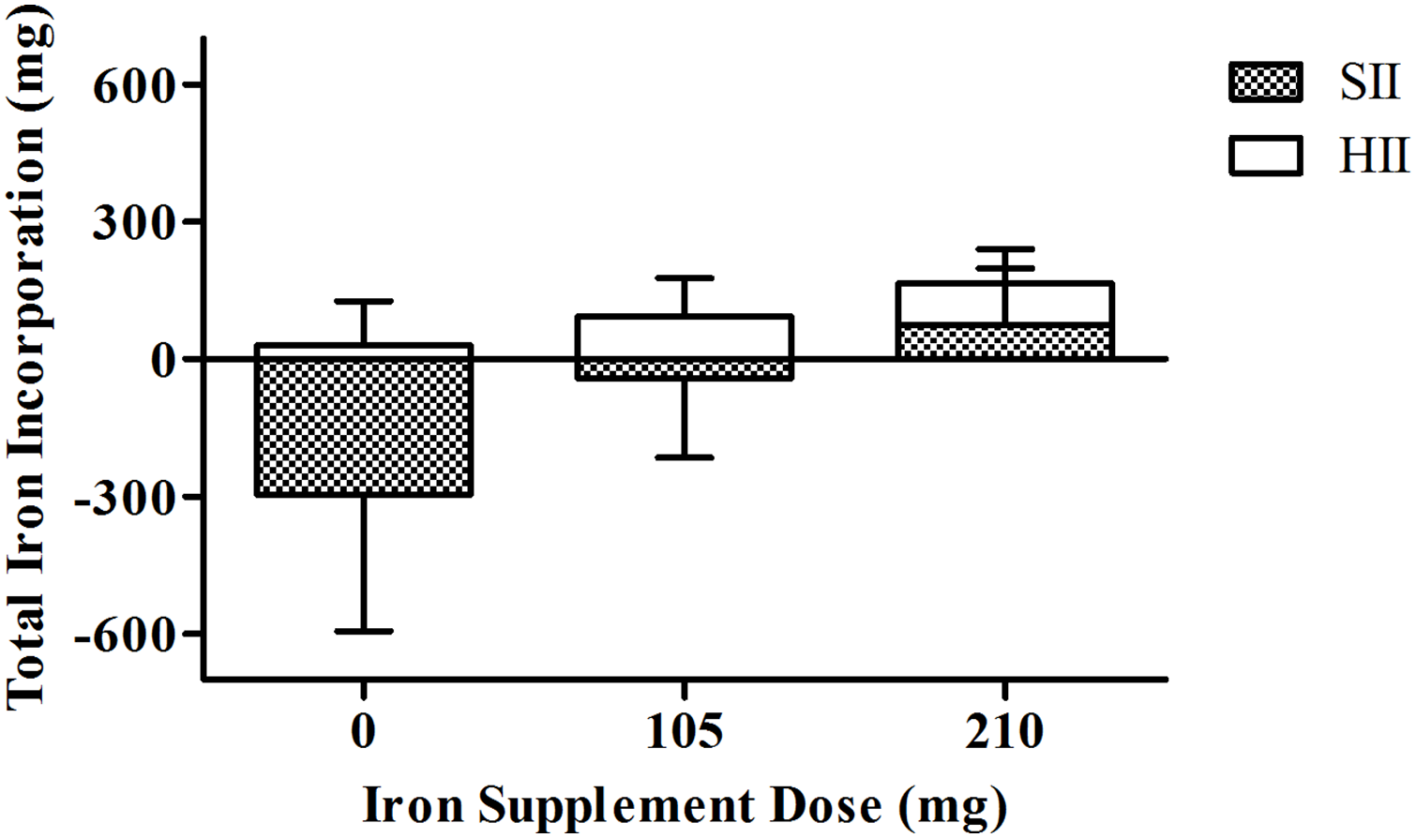
Iron incorporation into the iron storage (SII) and red cell compartment (HII) for each category of iron supplement dose [0 mg (*n* = 15), 105 mg (*n* = 144), 210 mg (*n* = 19)]. Overall, total iron incorporation (TII) was higher in iron-supplemented athletes compared with non-iron supplemented athletes.

Fig 1 compares the TII after altitude exposure in iron supplemented and non-iron supplemented athletes. Overall, TII was higher in athletes who were supplemented with either 105 mg or 210 mg of iron orally per day during altitude exposure compared with non-iron supplemented athletes. Specifically, iron supplementation of either 105 mg or 210 mg of oral iron per day during altitude exposure induced a large and very large increase in TII (0 *versus* 105 mg: *d* = 1.52 [0.95, 2.07]; 0 *versus* 210 mg: *d* = 2.13 [1.24, 2.92]).

## Discussion

This study found that prolonged, moderate altitude exposure (median elevation: 3,000 m; median duration: 21 days) enhanced erythroid iron incorporation and Hb_mass_ in iron supplemented athletes. Moreover, daily oral iron supplementation attenuated the reduction in serum ferritin levels following altitude exposure, compared with non-iron supplemented athletes. Therefore, we conclude that daily oral iron supplementation may support Hb_mass_ production and maintain iron balance in athletes with low pre-altitude serum ferritin levels during moderate altitude exposure.

### Hb_mass_ Response to Moderate Altitude

In the current study, moderate altitude exposure enhanced Hb_mass_ by ~3–4% in oral iron supplemented athletes. These findings support Gore et al. [30], who calculated that an individual athlete exposed to moderate altitude >2,200 m for 300 h might expect a mean increase in Hb_mass_ of ~3.2%. In contrast, non-iron supplemented athletes in the current study did not enhance their Hb_mass_ beyond the typical error of measurement of the CO rebreathing technique (~1.8%) [30]. We speculate that oral iron supplementation may have supported further Hb_mass_ production in these non-iron supplemented athletes. However, it is also possible that poor intestinal iron absorption and/or erythroid iron uptake also limited iron incorporation by erythroid precursor cells, thereby compromising Hb_mass_ production in these athletes. Therefore, although our findings largely suggest that oral iron supplementation supports Hb_mass_ production in athletes undertaking moderate altitude exposure, further research is required to establish a clear serum ferritin threshold to guide oral iron supplementation in athletes.

Our findings that moderate altitude exposure enhances Hb_mass_ by 3–4% in iron-supplemented athletes contrast those of the Monte Carlo simulation by Rasmussen, Siebenmann, Diaz & Lundby [31]. These authors concluded altitude exposure must exceed two weeks at an altitude of >4,000 m to increase red cell volume. However, Garvican-Lewis, Halliday, Abbiss, Saunders & Gore [32] recently demonstrated a 3.1% increase in Hb_mass_ in distance runners following three weeks exposure to 1,800 m. We speculate that differences in oral iron supplementation practices could explain some of the discrepancies between our findings and those of Rasmussen, Siebenmann, Diaz & Lundby [31]. Specifically, whereas 72% of the athletes analysed by Rasmussen, Siebenmann, Diaz & Lundby [31] were not iron supplemented, 92% of athletes in our study ingested oral iron supplements. Therefore, routine oral iron supplementation may ensure athletes are able to support erythropoiesis when exposed to moderate altitude exposure for two weeks or more.

### Total Iron Incorporation & Iron Parameter Responses

We estimated the amount of the iron mobilised from the iron storage compartment and directed to the erythron based on the change in Hb_mass_ and serum ferritin levels following moderate altitude exposure. In general, oral iron supplemented athletes incorporated more iron into the erythron and mobilised less iron from the iron storage compartment compared with non-iron supplemented athletes. Therefore, oral iron supplementation may assist athletes to maintain a healthy iron balance at altitude, in turn ensuring sufficient iron is available to support accelerated erythropoiesis at altitude.

Athletes supplemented with 210 mg of oral iron per day increased their serum ferritin levels following moderate altitude exposure, despite a large Hb_mass_ response. Such an observation indicates the oral iron supplement dose exceeded bone marrow iron uptake in these athletes resulting in the storage of excess iron. One explanation for this observation is that the iron absorption mechanisms were already up-regulated before altitude exposure owing to these athletes presenting as Stage 1 iron deficient. Iron deficiency and altitude exposure both result in systemic hypoxia and stimulate similar changes in iron metabolism. Two such changes in iron metabolism are an increase in HIF-2α transcription [33,34] and hepcidin suppression [35–37]. Hepcidin suppression is a favourable response to hypoxia that improves iron export from storage cells by reducing the degradation of ferroportin iron export channels located on the cellular surface of iron storage cells. Simultaneously, HIF-2α increases the expression of ferroportin [34] and proteins involved in iron absorption (i.e. divalent-metal transporter-1, duodenal cytochrome B) [38]. Finally, the hypoxic stabilisation of HIF-1α enhances the expression of iron transport proteins (i.e. TfR-1) [39]. These changes in iron absorption and transport mechanisms in iron deficient athletes during altitude exposure may explain why oral iron supplementation effectively replenished the iron storage compartment in athletes who ingested 210 mg of oral iron supplements per day.

In contrast to iron-supplemented athletes, non-iron supplemented athletes mobilised a large amount of iron from storage sites during altitude exposure, but incorporated less iron into the erythron. Such a response may indicate impaired iron delivery to the erythron, since these athletes had otherwise healthy serum ferritin levels before altitude exposure. Although not measured in the current study, an exercise-induced increase in hepcidin during exercise recovery in non-supplemented athletes may have temporarily inhibited iron export from reticuloendothelial macrophages, thus limiting its delivery to the erythron during the post-exercise recovery period. In support of this hypothesis, Peeling et al. [40] showed that athletes with high pre-exercise serum ferritin levels (>100 μg·L^-1^) at sea level exhibit a significantly greater post-exercise hepcidin response 3 h following interval exercise than athletes with low serum ferritin levels (serum ferritin <30 μg·L^-1^). A similar response may also occur during LHTL, despite hepcidin suppression at altitude [35], thus temporarily decreasing iron delivery to the bone marrow during the exercise recovery. However, the post-exercise hepcidin response during LHTL is yet to be investigated.

### Serum Ferritin Threshold for Iron-Supplementation at Moderate Altitude

To date, no clear serum ferritin threshold exists to guide oral iron supplementation for athletes planning an altitude sojourn. At sea level, athletes with serum ferritin levels <35 μg·L^-1^ are often prescribed oral iron supplements to restore and maintain iron balance [10]. A higher serum ferritin threshold is likely required for athletes undertaking altitude exposure to compensate for an hypoxic-mediated increase in erythroid iron uptake at altitude. In the absence of clear criteria, we supplemented both males and females with serum ferritin levels <100 μg·L^-1^. We based our rationale on the findings of Brugnara, Chambers, Malynn, Goldberg & Kruskall [41], who showed healthy subjects with serum ferritin levels <100 μg·L^-1^ developed a functional iron deficiency when undergoing synthetic EPO administration. We acknowledge that synthetic EPO administration bypasses an hypoxic-mediated increase in HIF-1α and HIF-2α transcription during prolonged altitude exposure. Therefore, some athletes may have sufficient iron stores to support erythropoiesis during moderate altitude exposure despite serum ferritin levels <100 μg·L^-1^, since HIF activation stimulates the expression of proteins involved in intestinal iron absorption and iron export from reticuloendothelial macrophages. Based on our results, we are unable to define a specific serum ferritin threshold at which to commence oral iron supplementation in athletes undertaking prolonged altitude exposure. Thus, further research is required to establish altitude-specific serum ferritin thresholds to guide oral iron supplementation during moderate altitude exposure.

Some disadvantages of oral iron supplementation include side effects such as nausea, flatulence and gastrointestinal discomfort. Furthermore, oral iron supplements transiently increases oxidative stress on the gut mucosa and may disrupt the gut microbacteria [42]. Hence, if oral iron supplements are not well-tolerated, then parenteral iron supplementation may be indicated to increase an athlete’s iron stores in preparation for altitude exposure [9]. However, parenteral iron supplementation should be considered in consultation with a trained sports physician. In light of our findings, the development of altitude-specific, iron supplementation guidelines could help to optimise athletes’ hematological adaptations to prolonged altitude exposure. Furthermore, refinement of iron supplement guidelines for athletes undertaking altitude exposure may also help to avoid the health risks associated with unnecessary oral iron supplementation in athletes with otherwise healthy iron stores.

### Limitations

This study has several limitations. Firstly, we were unable to analyse athletes’ training volume or dietary iron intake as covariates in our statistical model. We recommend future studies account for these factors to determine their influence on the Hb_mass_ and iron parameter response to altitude exposure. Secondly, exercise and/or illness may have increased inflammatory cytokines at altitude, in turn increasing serum ferritin levels owing as part of the acute phase response [43]. In the current study, venous blood samples were collected before exercise under standardised conditions. Therefore, we believe it is unlikely that an exercise-related increase in inflammatory cytokines influenced the blood iron parameter levels reported here. However, we cannot exclude the possibility than an IL-6 mediated increase in hepcidin during post-exercise recovery may have compromise intestinal iron absorption and/or iron recycling from reticuloendothelial macrophages. Thirdly, the low sample size of the non-iron supplemented and 210 mg oral iron supplement group meant we were unable to extend our model to consider whether sport discipline and altitude duration moderated the Hb_mass_ response to altitude exposure. Thus, these factors may also have influenced the Hb_mass_ response to moderate altitude exposure reported here. However, the influence of altitude duration on the Hb_mass_ response to moderate altitude has previously been analysed by Gore et al. [30]. Finally, given that prolonged altitude exposure enhances several iron-dependent oxidative enzymes, such as cytochrome C oxidase and citrate synthase, it is likely that some of the iron derived from oral iron supplements was directed to the mitochondria to support the synthesis of these iron dependent non-haem proteins.

## Conclusion

Two to four weeks of low-moderate altitude exposure (1,350–3,000 m) increased Hb_mass_ in well-trained iron supplemented athletes. Daily oral iron supplementation improved erythroid iron incorporation and resulted in higher serum ferritin levels in athletes who ingested 105 mg and 210 mg of oral iron supplements per day, relative to non-supplemented athletes. We conclude that oral iron supplementation likely supports Hb_mass_ production during moderate altitude exposure, and may help to replenish the iron storage compartment in some iron deficient athletes.
